# Ocean Acidification May Aggravate Social-Ecological Trade-Offs in Coastal Fisheries

**DOI:** 10.1371/journal.pone.0120376

**Published:** 2015-03-17

**Authors:** Rudi Voss, Martin F. Quaas, Jörn O. Schmidt, Ute Kapaun

**Affiliations:** 1 Department of Economics, Christian Albrechts Universitat zu Kiel, Wilhelm-Seelig-Platz 1, Kiel, Germany; 2 Kiel Institute for the World Economy, Kiel, Germany; Swedish University of Agricultural Sciences, SWEDEN

## Abstract

Ocean Acidification (OA) will influence marine ecosystems by changing species abundance and composition. Major effects are described for calcifying organisms, which are significantly impacted by decreasing pH values. Direct effects on commercially important fish are less well studied. The early life stages of fish populations often lack internal regulatory mechanisms to withstand the effects of abnormal pH. Negative effects can be expected on growth, survival, and recruitment success. Here we study Norwegian coastal cod, one of the few stocks where such a negative effect was experimentally quantified, and develop a framework for coupling experimental data on OA effects to ecological-economic fisheries models. In this paper, we scale the observed physiological responses to the population level by using the experimentally determined mortality rates as part of the stock-recruitment relationship. We then use an ecological-economic optimization model, to explore the potential effect of rising CO_2_ concentration on ecological (stock size), economic (profits), consumer-related (harvest) and social (employment) indicators, with scenarios ranging from present day conditions up to extreme acidification. Under the assumptions of our model, yields and profits could largely be maintained under moderate OA by adapting future fishing mortality (and related effort) to changes owing to altered pH. This adaptation comes at the costs of reduced stock size and employment, however. Explicitly visualizing these ecological, economic and social tradeoffs will help in defining realistic future objectives. Our results can be generalized to any stressor (or stressor combination), which is decreasing recruitment success. The main findings of an aggravation of trade-offs will remain valid. This seems to be of special relevance for coastal stocks with limited options for migration to avoid unfavorable future conditions and subsequently for coastal fisheries, which are often small scale local fisheries with limited operational ranges.

## Introduction

Accumulated anthropogenic carbon dioxide in the atmosphere dissolves in marine water to lower the pH, a process termed ocean acidification. About a third of excess CO_2_ in the atmosphere will accumulate in ocean waters [1–3]. Correspondingly, global surface pH has already decreased by more than 0.1 units since preindustrial times and is projected to lead to an additional drop in pH of 0.4 units (corresponding to a partial pressure of CO_2_ in seawater in micro-atmosphere of ~ 1000μatm) globally by the year 2100 and up to 0.8 units (pCO_2_ ~ 2000μatm) by the year 2300 [1, 3, 4]. Locally the effects can be even more severe. In coastal upwelling regions pCO_2_-values above 4000μatm could be reached in the future [5].

Ocean acidification will affect primarily calcifying organisms like mollusks, and forecasted ecological and economic impacts are severe [6]. Other recent biological studies have investigated the responses of fish, especially their early life stages, to increasing CO_2_ conditions. The physiological responses of these young developmental stages are much more pronounced than for more mature stages because they lack specialized mechanisms for tolerating low pH. Experimental evidence for such effects is still scarce, but is becoming increasingly available. While some earlier studies failed to find effects [7], recent experiments have shown clear effects of acidification, including changes in behavior, reductions in otolith size and metabolism, as well as larval tissue damage leading to reduced growth and survival rates [4, 8].

While it is by no means easy to assess how these effects on individual fish translate into effects on the productivity of the entire fish stock, management needs to be prepared to account for the effects of OA in defining goals on all temporal, spatial, and hierarchical scales.

Research to provide information regarding the effects of OA for use in marine fisheries management is a new field. It is easy to argue that such work should include a focus on early life stage survival because it is a bottleneck for recruitment and hence stock productivity. Changes in vital rates caused by OA among these stages will therefore challenge fisheries management in its objective to reach the triple bottom lines [9] of respecting ecological, economic and social needs (and especially in finding a healthy balance among these needs more generally).

Trade-offs between these 3 bottom lines do not only exist for fisheries, but also on the global scale. In particular, short-run economic objectives are more important than respecting the ecological objective of reducing global CO_2_ output. In this paper we argue that this failure to meet the triple bottom lines at the global level changes the boundary conditions for regional cases and makes it more difficult to meet the triple bottom lines in local ecosystem management.

Here, we use Norwegian Coastal Cod, one stock component of the ecologically and economically important Atlantic cod, *Gadus morhua*, as a case study to tentatively link physiological processes found in experimental studies [4] to dynamics at the population level. Atlantic cod forms several populations across the North Atlantic. It has a wide distribution with significant commercial importance dating back to at least medieval times. The high levels of exploitation have caused stock collapses in some regions [10] with strong ecological, economic and social consequences. We study stock dynamics and management of Norwegian Coastal Cod under conditions of increasing CO_2_ concentrations in marine water. For this particular stock, experimental evidence [4] suggests that OA adds another source of mortality to the already exploited stocks by reducing the recruitment success. The population is mainly distributed in the fjords along the Norwegian coastline and in the area of Lofoten. These high latitudes are predicted to be severely affected by future ocean acidification, with pH values reaching 7.7 [11–14] or even lower [15] by 2100 in most areas of the Arctic Ocean.

Norwegian coastal cod mainly supports a traditional, coastal, small-scale fishery. This fishery will be especially threatened because of the severity of impacts from local climate change. Its restricted spatial mobility further reduces adaptive potential. Data from analytical stock assessments are available [16].

The most important reason for considering Norwegian coastal cod is that published experimental data on changes in larval mortality rates under increasing ocean acidification are available for this stock [4]. Mindful of the lack of progress in reducing the production of CO_2_, globally, we use an ecological-economic model [17] to explore the potential effect of increasing CO_2_ levels on optimal management of Norwegian coastal cod. We quantify trade-offs between different management objectives, i.e. changes in harvest (as relevant for consumers), stock size (ecology), profits (capital holder), as well as fishing effort (a proxy for employment possibilities). This framework could be extended for incorporating biological and ecological information on the effects of OA not yet available. The scenarios considered here (as based on information from experimental research) include extreme CO_2_ conditions (i.e., well beyond projected future conditions). However, our focus is to explore the long-term (steady state) outcome and associated strategic management goals within the limitations imposed by the model assumptions and current data availability. Despite large uncertainties associated with the up-scaling of scarce experimental data to the population level, we argue that the results provide valuable forewarnings for managers and decision-makers about future challenges and anticipated trade-offs that will have to be evaluated.

## Materials and Methods

Considering the potential impact of ocean acidification on fisheries requires scaling from physiological responses to processes at the levels of the population and ecosystem. This can be done by appropriately modifying the parameters of growth, mortality and reproduction in a single-species model [18]. Here we concentrate on the modification of the parameters of a modeled stock-recruitment relationship to be used in an ecological-economic optimization model (S1 Materials). The parameter values used in the age-structured fishery model are given in S1 Table.

Mortality during the recruitment process is made up of both density-independent and density-dependent effects. Ocean acidification causes severe tissue damage in the larvae of Norwegian Coastal Cod which is predicted to result in a higher larval mortality rate [4]. We assume that these effects will influence the density-independent mortality during the whole recruitment phase and that the severe tissue damages in the larvae will result in an increased mortality of exactly the same magnitude. Based on experimental results, Frommel et al. [4] predict a mean increase in larval mortality of 12% in a medium OA scenario (pCO_2_ of 1800μatm) and of 75% in an extreme upwelling OA scenario (4200μatm). Potential CO_2_ pressures used in the experiments (and subsequent modeling) will only be reached in the long-run and/or effect only parts of the population (in the case of upwelling). Our approach therefore develops a framework to include climate change effects, e.g. physiological OA effects, in long-term management goal setting, rather than providing short-term, tactical advice.

Translating the experimental results to changes in the stock-recruitment relationship (for details see S1 Materials) our model shows reduced peak recruitment estimates, as would be expected. Recruitment success is defined as that point of time when a year-class enters the fished population, i.e. at an age of 2 years in the case of Norwegian coastal cod. It is usually regulated by a number of interacting variables also affecting the life stages following the larval stage [19, 20]. Therefore, our approach has to be seen as a rough approximation, where we assume that ocean acidification acts as an additional factor, causing a systematic decline in recruitment, with additional variation caused by a large variety of additional factors, at least some of which are taken into account in a Monte-Carlo sensitivity analysis (S1 Materials).

## Results

**Fig 1:**
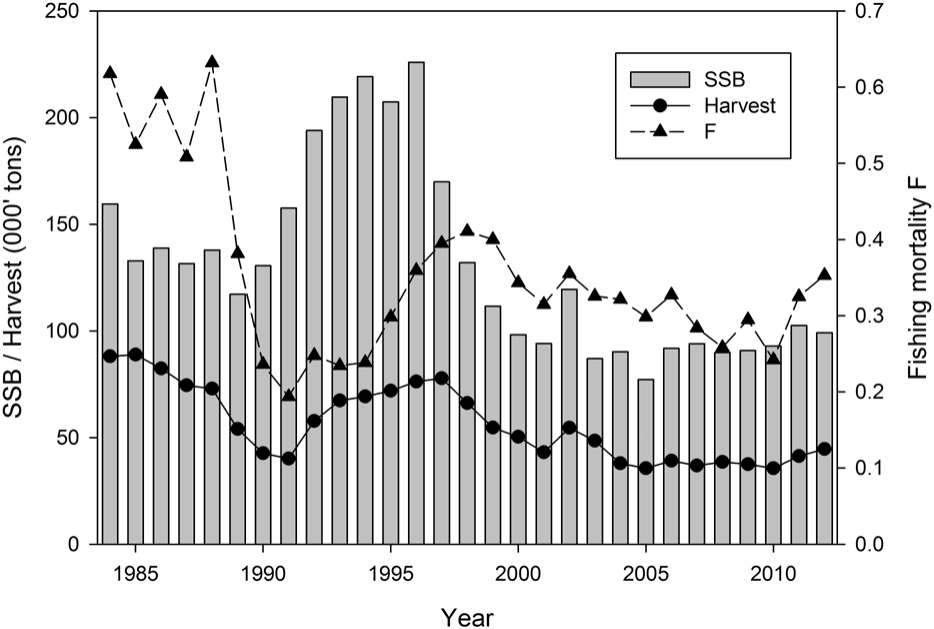
Stock dynamics. Spawning Stock Biomass (SSB), harvest and fishing mortality of Norwegian coastal cod.

Analytical assessment data are available since 1984 (Fig. 1), showing that the stock has been fished on a quasi-sustainable level in recent years (in spite of the possibility that realized fishing rates for cod are too high for sustainability at the ecosystem level in the long run [21]). Fishing mortality and spawning stock size have remained essentially stable since the early 2000s, with little fluctuation since.

We tested simulated outcomes for three fisheries management options under present day, medium, or high ocean acidification levels and the associated reduced recruitment rates. The management options include (i) retaining fishing rates at recent levels, (ii) optimal management, adjusted to status quo levels of OA, or (iii) optimal adaptation of management to ocean acidification. In this study, optimal management is defined as profit maximizing management, as profit maximization has been shown to go hand in hand with stock rebuilding for cod [22].

**Fig 2:**
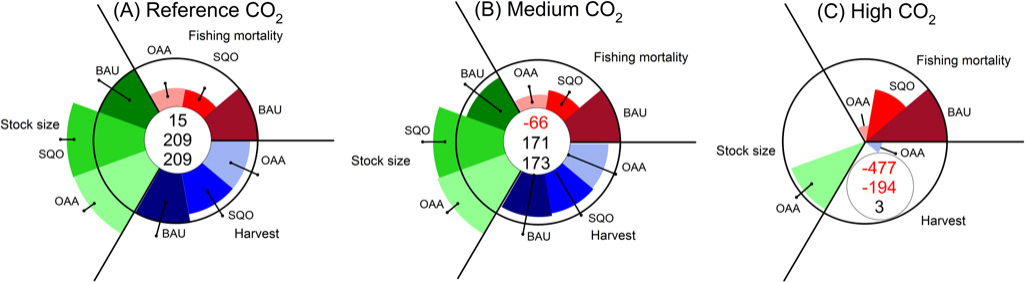
Summary of management options and trade-offs under increasing ocean acidification. Business as usual (BAU), Status Quo Optimum (SQO) as well as Optimal Adaptation to Acidification (OAA—see text for explanation) for 3 levels of ocean acidification. (A) Reference CO2. (B) Medium CO2 (1800μatm). (C) High CO2 (4200 μatm). Central numbers indicate total profits (million USD/year) for BAU, SQO and OAA management (top to bottom). Area of each pie slice is relative to business as usual, quasi sustainable values 2000–2012 (black circle), with error bars from sensitivity analysis.

In our model simulations, spawning stock biomass, harvest as well as profits decrease under increasing ocean acidification if the present day business-as-usual management is perpetuated (Fig. 2; Business As Usual, BAU).

Our model indicates, that the business as usual (BAU) fishery could turn unprofitable at 1800 μmol CO_2_ (Fig. 2b). Under the high CO_2_ scenario, the stock would collapse (Fig. 2c). Changing to an optimal management adapted to status quo CO_2_ conditions (Status Quo Optimum, SQO), might rebuild the stock, reduce the harvest slightly, but hold the potential of increasing the profits by a factor of 14 (+194 million USD; Fig. 2a) compared to BAU. With CO_2_ conditions at medium levels (Fig. 2b), our model supports the potential that the stock size, harvests and profits would all be reduced only slightly, under this management strategy. However, under conditions captured by our model, at high CO_2_ levels the stock would collapse. Only optimal adaptation to increasing CO_2_ conditions (Optimal Adaptation to Acidification, OAA) would stabilize the stock size even at 4200μatm CO_2_ (Fig. 2c). Under these conditions, tolerated sustainable fishing pressure would be very low, with small harvests and low levels of profit.

The veracity, accuracy, and precision of our conclusions obviously depend on the extent to which our model captures the reality of the complex systems involved in carrying out commercial fishing on cod in the regions we are studying. In spite of these caveats, however, certain conclusions are essentially inescapable. Our focus on specific management options highlights the existence of trade-offs between ecological-economic objectives on the one hand and social objectives on the other hand: Fishing effort, (including the work force needed and jobs provided by the fishery), has to be drastically reduced, in order to stabilize sustainable exploitation in order to account for the impacts of ocean acidification.

## Discussion

For the case of a regional small-scale fishery, this study demonstrates the strong likelihood of negative long-term effects of ocean acidification on the three bottom lines of fisheries management: ecological, economic and social objectives. Our study provides a showcase of how to use the results of laboratory experiments to explore implications at the population level of biological resources and illustrate what will have to be done in management. Our main interest lies in demonstrating the potential long-term effect of OA on fisheries and fish stocks. The relevant time scales of climatic change are long compared to the time scale at which fish population dynamics reach a steady state. For this reason we focus on the long-term effects of acidification and management on the fish population. Achieving stable and normal structure and function for the remainder of the ecosystem awaits further research.

Our approach can be used for other species in other locations. Transfer to other cases will require quantitative evidence from laboratory experiments showing reactions to OA. We have chosen the case of Norwegian Coastal Cod. This fishery mainly consists of small vessels (13–28 m length), managed by a non-transferable vessel quota [23]. As in many other small-scale fisheries [24], labor is usually paid to the crew in shares [23]. A small operational range leads to limited adaptability and makes the fishery vulnerable to climate change and its impacts on stock productivity. Therefore, as for global warming [25], adaptation of fisheries management to increasing OA is urgently needed to adapt to the unintended effects of anthropogenic CO_2_ production.

The full complexity of systems such as those we are dealing with can never be adequately captured in a model. Progress toward alleviating this constraint would involve explicit treatment of other measureable factors of known importance. In fact, it seems imperative that future research will look at combined effects (e.g. ocean warming and OA [26] together), in order to approach a more complete evaluation of the full extent of the imposed threats. Obviously, our results can be generalized to any stressor (or combination of stresses) known to result in reduced recruitment success. The main findings of an aggravation of trade-offs between ecological (stock size) and social (sustainable fishing efforts) objectives clearly remain valid. Laboratory studies, including the one which we take advantage of in this paper, are limited. In our case, laboratory studies (and our simulating of ocean acidification) included CO_2_ concentrations much higher than predicted (4). Both the laboratory studies and our model neglect all of what could be numerous genetic responses of cod to abnormal pH levels. The extended generation time of long-lived species such as cod would be an obvious factor in the realized pace of evolutionary adaptation with changes that might not become obvious or measurable for years. Our model set-up abstracts from real-world complexities in several other respects, so that the results express examples of what might happen rather than predictions: E.g. natural mortality rates are assumed constant over age, as in the ICES stock assessment on which we base the parameterization of the model, but contrary to empirical information from survivorship curves [27, 28]. Adult food intake, growth rates, behavior, and distribution are influenced by changes in pH. Because there is no quantitative information on these relationships available yet, they are also not reflected in the model. The same applies for ecosystem impacts, like e.g. prey abundance, or evolutionary aspects of ecosystem dynamics. Such factors can in principle be included in our modeling framework, but need to be quantified. Unfortunately, such quantification is not yet available.

Natural recruitment variability is usually high in marine fish species and might well mask declines due to increased CO_2_ ocean acidification, as our sensitivity analysis shows (Fig. 2). Increasing CO_2_ levels, however, can clearly be expected to reduce recruitment success in a systematic manner, and such changes will impact long-term ecological-economic aspects of the fishery. While some of these may be things that can be accounted for in large part by adaptive fisheries management, the impacts involving social factors are anything but trivial and trade-offs to be made in future fisheries management as related to ocean acidification are enormous.

Finally, we are mindful of the fact that our work merely considers the symptoms of a much larger problem. As with our work, all of the research undertaken to demonstrate the impact of increasing CO_2_ in the environment fails to find sustainability in the global rate at which this gas is produced anthropogenically. Unless this problem is addressed at the global scale, all of the resulting consequences, including ocean acidification with all of its ramifications (only several of which we have addressed), will continue to intensify.

## Supporting Information

S1 Materials Age-structured fishery model, stock-recruitment function, and sensitivity analysis. (DOCX)

S1 Table Parameter values used in the age-structured fishery model. (DOCX)
