# Supplementary material for: Ocean Acidification May Aggravate Social-Ecological Trade-Offs in Coastal Fisheries
Source: PLoS One. 2015 Mar 17;10(3):e0120376. doi: 10.1371/journal.pone.0120376 (PMC4363370; doi:10.1371/journal.pone.0120376)
Supplement: S1 Materials — (DOCX) [file pone.0120376.s001.docx]

**Materials S1: Age-structured fishery model, stock-recruitment function, and sensitivity analysis**

## Age-structured fishery model

We apply an age-structured ecological-economic optimization model [1, 2]. We use $x_{st}$ to denote the number of fish in age group *s* and at the beginning of year *t*. We use $\alpha_{s}>0$, $s=1,\ldots, n$ to denote age specific natural survival rates $\gamma_{s}>0$, $s=1,\ldots, n$, to denote age specific proportions of mature individuals and $w_{s}$, $s=1,\ldots, n$, to denote the mean weights (in kilograms) of fish in age group *s*, and $q_{s}$, $s=1,\ldots, n$, to denote the age-specific catchabilities. We assume the maximum age (n) to be 10 (Table S1). All of these parameters are assumed to be constant as in [1], and as in the standard biological stock assessments [3]. Using $F_{t}$to denote the instantaneous fishing mortality in year *t*, and *ø_1_* to denote the density-independent and *ø_2_* to denote the density-dependant parameters of a Ricker stock-recruitment function, the age-structured population model with harvesting activity can be summarized as:

We use age-specific survival rates, weight at age and age specific maturity from ICES for the year 2012. The age-specific catchability is calculated with the help of the instantaneous age-specific fishing mortality rates, normalizing the highest age-specific fishing mortality rate to one (1.0) and determining the catchability of different age classes in relation to the catchability of this age class. The resulting parameter values are listed in Table S1.

We are interested in the effect of ocean acidification (OA) on fish stocks. The relevant time scales of climatic change are long compared to the time scale at which fish population dynamics reach a steady state. For this reason we focus on a fish population in steady state. Assuming a steady-state, the equilibrium spawning stock biomass is obtained as

Here, we use χ_0_ to denote spawning stock biomass as consistent with earlier publications [4-7]. The underlying assumption was that spawning stock biomass is a measure of egg production in numbers, i.e. higher numbers of spawning stock biomass producing higher numbers of eggs.

We assume a fishing cost function of the Clark [8]-Spence [9] type (as in [10]) for the Baltic cod fishery). Profits are given by $\pi_{t}=p H_{t}-c F_{t}$, where $H_{t}=\sum_{s=1}^{n} q_{s}\left(1-e^{-F_{t}}\right)x_{st}$ is aggregate harvest in year t. We use the average price for Norwegian coastal cod for the period 1985-2000 from [11], $p=8.9$NOK/kg. To determine the cost parameter c, we use the estimate from [11] that the profit ratio is about 2.8% for Norwegian coastal cod. Hence, $c=0.972 p H_{t}/F_{t}$, and *π_t_ =* 0.028*pH_t_*.

According to empirical data [3], the average maximum of age-specific fishing mortalities in the period 2000-2012 was$F=0.55$. We use the population model to determine the equilibrium harvest at this fishing mortality, which is 55,232 tons$=0.0552$million tons. Using this, we obtain $c=0.971\times8.9\times0.0552/0.55=867$million NOK (Norwegian Kronor).

## Scaling from physiological responses to population processes

The primary focus of recent biological studies relates to the effects of ocean acidification on physiological processes. Considering the potential impact of ocean acidification on fisheries requires applying information about physiological responses at the levels of populations and ecosystems and their inherent processes. A simple way to accomplish this is to consider how ocean acidification might modify the parameters of growth, mortality and reproduction in a single-species model [12]. Here we concentrate on the modification of the parameters of the stock-recruitment relationship in an age-structured fishery model.

We assume that egg production in year *t*, $N_{0t}$, is proportional to spawning stock biomass, $x_{0t}$, i.e.$N_{0t}=f \text{x}_{0t}$, where *f* is the net fecundity in the population [13]. We assume that the stock-recruitment relationship is of the Ricker [14] type. This type of stock-recruitment relationship has been shown to be an appropriate description of recruitment biology of cod [15]. According to the Ricker model [14, 16], the development of the early-life history follows , where $N_{t}(0)=N_{0t}$, and recruits enter the fish stock at $\tau=1$, i.e. $x_{1.t+1}=N_{t}(1)$. Natural mortality is made up of three components (). Following Frommel et al. [17], ocean acidification causes severe tissue damages in the larvae which is likely to result in a higher larval mortality rate. This leads to a density-independent mortality rate *a* caused by acidification. Furthermore, *b* is the density-independent mortality rate at baseline conditions, and is the density-dependent mortality rate which increases with the spawning stock (e.g., because of cannibalism [16]). Solving the differential equation, we obtain

where . In the baseline-scenario, we have $a=0$, in the acidification scenarios, $e^{-a}$is the fraction of cod in the early life history stages that survives the effect of acidification. We use the data from experiments to quantify this effect.

To estimate the stock-recruitment relationship for the baseline scenario we used ICES [3] data for the Norwegian coastal cod for the years 1991 to 2012. Following [15], we assumed log-normal auto-correlated errors, and estimated parameters for the model

,

where $\xi_{t+1}=\nu\xi_{t}+\varepsilon_{t}$, and $\varepsilon_{t}$ is the random error. We obtained estimates with 95% confidence interval [-1.171;-0.197] and 6.20/million tons with 95% confidence interval [1.68; 10.73]/million tons.

## Sensitivity analysis

Error bars were determined with respect to the major source of parameter uncertainty, the standard errors of the parameters from the stock recruitment function. We performed a Monte-Carlo analysis as in [2].

To assess the parameter uncertainty with respect to stock-recruitment functions, we generated 10,000 random parameter values for , using the mean and variance as derived from the statistical estimation of this parameter. For each parameter set, we determined the spawning stock biomass, harvest and profits in the three fishing scenarios. The resulting values were assumed to be log-normally distributed, and corresponding error bars (i.e., point result times the exponential function of $\pm$one standard deviation) are obtained from the standard deviation of the sample of results.

**References**

1. Tahvonen O (2009) Economics of harvesting age-structured fish populations. Journal of Environmental Economics and Management 58: 281-299 (2009).
2. Tahvonen O, Quaas MF, Schmidt JO, Voss R (2013) Effects of species interaction on optimal harvesting of an age-structured schooling fishery. Environmental and Resource Economics 54: 21-39.
3. ICES Report of the Arctic Fisheries Working Group 2013 (AFWG) (2013) ICES CM 2013/ACOM:05 726pp.
4. Voss R, Hinrichsen H-H, Quaas MF, Schmidt JO and Tahvonen O (2011) Temperature change and Baltic sprat: from observations to ecological-economic modelling. ICES J Mar Sci 68(6): 1244-1256.
5. Tahvonen O, Quaas MF, Schmidt JO, Voss R. (2013) Optimal harvesting of an age-structured schooling fishery. Environ Res Econ 54(1): 21-39.
6. Voss R, Quaas MF, Schmidt JO, and Hoffmann J. (2014) Regional trade-offs from multispecies maximum sustainable yield (MMSY) management options. Mar Ecol Prog Ser 498: 1-12.
7. Voss R, Quaas MF, Schmidt JO, Tahvonen O, Lindegren M, Möllman C (2014) Assessing social-ecological trade-offs to advance ecosystem-based fisheries management. PLoS ONE 9(9): e107811.
8. Clark CW (1990) Mathematical Bioeconomics. 2nd Edition Wiley, New York.
9. Spence AM (1974) Blue whales and applied control theory. In H. W. Gottinger (ed.), System Approaches and Environmental Problems (pp. 97–124) Göttingen: Vandenhoeck and Ruprecht.
10. Quaas MF et al. (2012) Fishing Industry Borrows from Natural Capital at High Shadow Interest Rates. Ecological Economics 82: 45–52.
11. McWhinnie SF (2007) The Impact of Rights-Based Management Regimes on Fishery Productivity, Proceedings of ESAM07, paper 125, University of Queensland.
12. Le Quesne WJF, Pinnegar JK (2012) The potential impacts of ocean acidification: scaling from physiology to fisheries. Fish and Fisheries 13: 333-344.
13. Hilborn R, Walters CJ (1992) Quantitative GFish Stock Assessment – Choice, Dynamics and Uncertainty. Kluwer Academic Publishers.
14. Ricker WE (1954) Stock and recruitment. Journal of the Fisheries Research Board of Canada 11: 559-623.
15. Cook RM, Sinclair A, Stefánsson G (1997) Potential collapse of North Sea cod stocks. Nature, 385: 521-522.
16. Quinn TJ, Deriso RB (1999) Quantitative Fish Dynamics. Oxford University Press.
17. Frommel AY, Maneja R, Lowe D, Malzahn AM, Geffen AJ, Folkvord A, Piatkowski U, Reusch TBH, Clemmesen C (2012) Severe tissue damage in Atlantic cod larvae under increasing ocean acidification. Nature Climate Change 2: 42–46.
