# Supplementary material for: Ocean Acidification May Aggravate Social-Ecological Trade-Offs in Coastal Fisheries
Source: PLoS One. 2015 Mar 17;10(3):e0120376. doi: 10.1371/journal.pone.0120376 (PMC4363370; doi:10.1371/journal.pone.0120376)
Supplement: S1 Table — (DOCX) [file pone.0120376.s002.docx]

Table S1. Parameter values used in the age-structured fishery model.

| Age | Weight | Survival | Maturity | Catchability |
| --- | --- | --- | --- | --- |
| 1 | 0 | 1 | 0 | 0 |
| 2 | 0.336 | 0.819 | 0 | 0.002 |
| 3 | 1.038 | 0.819 | 0.02 | 0.045 |
| 4 | 2.006 | 0.819 | 0.16 | 0.169 |
| 5 | 2.998 | 0.819 | 0.46 | 0.377 |
| 6 | 3.727 | 0.819 | 0.69 | 0.81 |
| 7 | 4.783 | 0.819 | 0.87 | 0.915 |
| 8 | 5.071 | 0.819 | 0.91 | 1 |
| 9 | 5.851 | 0.819 | 0.96 | 0.975 |
| 10 | 7.446 | 0.819 | 1 | 0.975 |
